# Chronic preoperative steroid use and total shoulder arthroplasty: a propensity score matched analysis of early-onset infectious outcomes

**DOI:** 10.1186/s42836-024-00296-6

**Published:** 2025-03-05

**Authors:** Ekrem M. Ayhan, Aaron J. Marcel, Jacob M. Johnson, Richard S. Feinn, Karen M. Myrick

**Affiliations:** Frank H. Netter MD School of Medicine, Quinnipiac University, 370 Bassett Rd, North Haven, CT 06473 USA

**Keywords:** Total shoulder arthroplasty, Corticosteroids, Infection, Readmission, Propensity score matching

## Abstract

**Background:**

While the role of chronic preoperative steroid use in orthopedic outcomes has been studied, particularly in hip, knee, and lumbar surgeries, its impact on total shoulder arthroplasty (TSA) outcomes is not well understood. This study aimed to evaluate the impact of chronic preoperative steroid use on early-onset postoperative infectious outcomes and readmission within 30 days following TSA compared to patients without chronic steroid use.

**Methods:**

A retrospective analysis was performed using data from the American College of Surgeons National Surgical Quality Improvement Program (ACS-NSQIP) spanning from 2010–2018. Clinical data including preoperative demographics, operative variables, and 30-day post-TSA outcomes were collected. Groups were balanced using propensity score matching based on gender, age, race, ethnicity, BMI, functional status, ASA, smoking status, alcohol use, year of operation, and comorbidities. A conditional logistic regression model was used to calculate odds ratios for each outcome measure.

**Results:**

A total of 3,445 identified cases were included in this analysis after propensity score matching, with 1,157 exhibiting chronic steroid use. The steroid group demonstrated significantly greater rates of readmission (OR: 1.86; 95% CI: 1.22–2.81; *P* = 0.004). No significant differences were observed between groups in all other adverse outcomes, including reoperation, specific infectious outcomes, and combined infectious outcomes.

**Conclusions:**

Preoperative chronic steroid use is an independent predictor of readmission but not infection following TSA. While the surgeon should be aware of the increased risk of readmission associated with chronic steroid use, the role of steroid use as a risk factor for postoperative infections following TSA may be less pronounced, particularly compared to surgeries of other joints. Further investigation of infectious outcomes and readmissions with longer follow-up is needed to clarify the specific role of chronic preoperative steroid use in adverse outcomes following TSA.

## Background

Total shoulder arthroplasty (TSA) is a gold-standard surgical intervention for patients with degenerative or inflammatory conditions of the shoulder. This procedure encompasses two main types: anatomic (aTSA) and reverse (rTSA). Currently, rTSA generally predominates as the primary surgical option, particularly in patients with extensive wear and deformity of the glenohumeral joint, while aTSA remains a viable option in patients with intact rotator cuffs [1].

Recent advances in implant design, surgical technique, and postoperative treatment for both aTSA and rTSA have led to significant growth in TSA incidence over the last two decades [2]. From 2012 to 2017, the population-adjusted incidence of primary rTSA increased from 7.3 to 19.3 cases per 100,000 people, while the incidence of aTSA increased from 9.5 to 12.5 cases per 100,000 people [3]. Additionally, from 2009–2018, the overall prevalence of glucocorticoid use in the United States increased significantly from 6.4% to 7.7% [4]. This necessitates updated preoperative risk assessments to reduce morbidity and minimize adverse outcomes following TSA, particularly for the patient using corticosteroids.

Corticosteroids modulate gene expression by inhibiting transcription and translation of inflammatory cells [5]. This anti-inflammatory mechanism and immunosuppression may contribute to adverse outcomes, including surgical site infections, pneumonia, sepsis, shock, reoperation, and readmission [6]. A recently described mechanism for the adverse outcomes related to chronic preoperative steroid use is oxidative stress [7]. Long-term preoperative steroid use has been shown to paradoxically increase systemic oxidative stress [8]. Thus, chronic preoperative steroid use combined with the physiological stress induced by surgery may contribute to a reactive oxygen species (ROS)-mediated increase in susceptibility to adverse outcomes following orthopedic surgery.

Analyses assessing the relationship between chronic steroid use and TSA complications are limited, with one study reporting that only readmission was associated with chronic steroid use after controlling for significant covariates [9]. However, strong evidence supports that the risk of complications is associated with chronic steroid use in other orthopedic surgeries [10]. Currently, preoperative steroid management is individualized based on the patient’s history of glucocorticoid intake and likelihood of hypothalamic–pituitary–adrenal (HPA) axis suppression [11]. As TSA incidence increases, an updated understanding of the risks associated with chronic preoperative steroid use is critical for the shoulder surgeon. Further research that carefully controls for covariates can help elucidate specific factors that may predispose chronic steroid users to adverse outcomes following TSA. Thus, the purpose of this study was to evaluate the impact of chronic preoperative steroid use on early-onset postoperative infectious outcomes and readmission within 30 days following TSA.

## Methods

### Data source

The American College of Surgeons National Surgical Quality Improvement Program (ACS-NSQIP) is a validated surgical outcomes registry that prospectively obtains clinical data, including preoperative patient demographics, operative variables, and 30-day postoperative outcomes. The NSQIP database allows for stratification of data based on various procedures using Current Procedural Terminology (CPT) codes [12]. Thus, the NSQIP database is valuable for high-powered analyses of specific surgical outcomes. Additionally, the NSQIP database is believed to be more accurate than similar surgical complications databases [13–15].

### Data collection

This retrospective review of the NSQIP database used CPT code 23,472 to identify all cases of aTSA/rTSA between 2010–2018. The “STEROID” variable was used to determine the two cohorts: “No Steroid User” and “Steroid User.” Steroid user is defined by the NSQIP database as any patient who has required the regular administration of oral or parenteral corticosteroids (i.e., Prednisone, Decadron) or immunosuppressant medications for a chronic medical condition (i.e., chronic obstructive pulmonary disease (COPD), asthma, rheumatological disease, rheumatoid arthritis, inflammatory bowel disease) within 30 days prior to the index operation or at the time that the patient is being considered for surgery. Demographics, comorbidities, and 30-day postoperative outcomes were compared between these groups.

Patient characteristics included age, sex, race, and body mass index (BMI). Patient comorbidities included functional status, smoking, ASA classification score, diabetes mellitus, hypertension, COPD dyspnea, congestive heart failure, bleeding disorders, renal failure, and operation time. Infectious outcomes included surgical site infections, deep incisional infections, organ space infections, pneumonia, urinary tract infections, sepsis, and septic shock. All these infectious outcomes were also analyzed together to compare the incidence of any infection. Other outcomes included reoperation and readmission (Table 1).

**Table 1:** Characteristics of patients treated with aTSA/rTSA, categorized by chronic preoperative steroid use

Variable	No Steroid Use	Steroid Use	Standardized Difference*
*n*	2,288	1,157	
Age (Years)	68.9 ± 10.4	68.1 ± 10.5	0.076
Sex (Female)	1,475 (64.5%)	797 (68.9%)	0.093
Race (White)	1,969 (86.1%)	934 (80.7%)	0.147
BMI^a^	29.8 ± 6.4	30.1 ± 6.9	0.045
Functional Status (Dependent)	81 (3.6%)	41 (3.6%)	0.000
Smoker (Within 1 year)	230 (10.1%)	127 (11.0%)	0.030
ASA^b^ Classification Score	2.7 ± 0.6	2.8 ± 0.5	0.186
Diabetes	346 (15.1%)	182 (15.7%)	0.017
Hypertension	1,551 (67.8%)	776 (67.1%)	0.015
COPD^c^	268 (11.7%)	167 (14.4%)	0.082
Dyspnea	279 (12.2%)	157 (13.6%)	0.041
Congestive heart failure	14 (0.6%)	18 (1.6%)	0.098
Bleeding Disorders	114 (5.0%)	55 (4.8%)	0.011
Renal Failure	24 (1.0%)	9 (0.8%)	0.028
Operation Time (Min)	114.3 ± 46.2	106.4 ± 50.5	0.169

^*^Standardized difference where < 0.2 was considered small

^a^BMI = Body Mass Index, ^b^ASA = American Society of Anesthesiologists, ^c^COPD = Chronic obstructive pulmonary disease

### Statistical analysis

All data were analyzed using descriptive and comparative statistics. An independent samples *t*-test was used for continuous variables. Pearson’s chi-square or Fischer’s exact test was employed, whenever appropriate, for categorical variables. Propensity score matching utilizing a nearest neighbor within-caliper strategy was used to control for baseline preoperative differences between the two cohorts, including both modifiable and non-modifiable risk factors. Matching was performed based on the following variables: gender, age, race, ethnicity, BMI, functional status, ASA, smoking status, alcohol use, year of operation, and comorbidities (diabetes, dyspnea, COPD, ascites, history of congestive heart failure, coronary artery disease, peripheral vascular disease, renal failure, weight loss, bleeding disorders, history of blood transfusion, and hypertensive medication use). Matching involved two controls (no steroid use) for each case (steroid use) using a caliper width of 0.01 on the propensity score. Of the 1,157 cases, virtually all (1,131, 97.8%) were successfully matched with two controls, with the remaining 26 matched to one control. Balance on baseline demographic and clinical features between groups was assessed with standardized difference, where a difference of less than 0.20 is considered comparable. The conditional logistic regression model used the matched groupings as strata and steroid use as the predictor variables to calculate odds ratios for each outcome. All statistical analyses were performed using the Statistical Package for Social Sciences (SPSS) 29 and statistical significance was set at an alpha level of 0.05.

## Results

This review of the NSQIP database for aTSA/rTSA yielded 23,581 operations from 2010–2018. Of these cases, steroid use for chronic conditions resulted in 1,157 patients for the “Steroid User” group, which were matched, using propensity scores, with 2,288 patients for the “No Steroid User” group.

The two groups were well-balanced on all demographic and clinical characteristics. No significant differences were observed between cohorts in any of the infectious outcomes, and in all infectious outcomes together (Table 2). The incidence of infectious complications was similarly low in both cohorts, with 2.8% and 2.3% experiencing any infectious outcome in the steroid group and no-steroid group, respectively (OR = 1.21, 95%CI = 0.78–1.87, *P* = 0.400). When comparing other outcomes, the steroid group was significantly more likely to be readmitted to the hospital within 30 days of the index operation than its no-steroid counterpart (OR = 1.86, 95%CI = 1.22–2.81, *P* = 0.004). Reoperation rates were low in both groups and did not differ significantly (Table 3).

**Table 2:** Conditional logistic regression model for infectious outcomes in patients treated with aTSA/rTSA based on chronic steroid use

Outcome	Occurrences	Odds Ratio	95% CI ^a^	*P*-Value
Surgical Site Infection		1.50	0.34–6.70	0.596
No Steroid Use	4 (0.2%)			
Steroid Use	3 (0.3%)			
Deep Incisional Infection		0.67	0.07–6.41	0.725
No Steroid Use	3 (0.1%)			
Steroid Use	1 (0.1%)			
Organ Space Infection		2.00	0.13–31.98	0.624
No Steroid Use	1 (< 0.1%)			
Steroid Use	1 (0.1%)			
Pneumonia		1.07	0.45–2.52	0.883
No Steroid Use	15 (0.7%)			
Steroid Use	8 (0.7%)			
Urinary Tract Infection		1.21	0.67–2.22	0.528
No Steroid Use	28 (1.2%)			
Steroid Use	17 (1.5%)			
Sepsis		0.60	0.17–2.18	0.438
No Steroid Use	10 (0.4%)			
Steroid Use	3 (0.3%)			
Septic Shock		2.00	0.40–9.91	0.396
No Steroid Use	3 (0.1%)			
Steroid Use	3 (0.3%)			
Any Infection		1.21	0.78–1.87	0.400
No Steroid Use	53 (2.3%)			
Steroid Use	32 (2.8%)			

^a^CI = Confidence interval

**Table 3:** Conditional logistic regression model for other outcomes in patients treated with aTSA/rTSA based on chronic steroid use

Outcome	Occurrences	Odds Ratio	95% CI ^a^	*P*-Value
Reoperation		1.89	0.97–3.67	0.060
No Steroid Use	18 (0.8%)			
Steroid Use	17 (0.5%)			
Readmission		1.86	1.22–2.81	0.004
No Steroid Use	46 (2.0%)			
Steroid Use	44 (3.8%)			

^a^CI = Confidence interval

## Discussion

In this propensity score matched analysis, we reported chronic preoperative steroid use to be an independent risk factor for readmission but not infection within 30 days of surgery. To our knowledge, this study was the first to employ propensity score matching to evaluate infectious outcomes following TSA in patients with chronic preoperative steroid use. A recent review of the NSQIP database by Fassler et al. analyzed 39,876 patients who underwent arthroscopic rotator cuff repair (aRCR) and found that chronic preoperative steroid use was not an independent risk factor for infectious outcomes following aRCR [16]. Although the procedures differ, aRCR and TSA patients generally share similar comorbidity profiles, and overall infection rates are similar as well [17–19]. Another review of the NSQIP database by Ling et al. found that chronic preoperative steroid use is not an independent predictor of infectious outcomes within 30 days following TSA. Additionally, like the present study, this analysis found readmission to be associated with chronic preoperative steroid use [10]. Importantly, however, the present study employed propensity score matching rather than multivariable analyses to control for baseline characteristics. Propensity score matching is advantageous in a study of this design, since there are several reasons for which patients may be taking steroids preoperatively, and this approach balances covariates by creating matched groups before analyzing the effect [20–23]. These consistent findings, despite differing statistical methodology, suggests that there may be a distinct relationship between chronic preoperative steroid use and infectious outcomes in surgeries of the shoulder [24].

The broader literature suggests that chronic steroid users undergoing orthopedic surgeries have a heightened susceptibility to infections [7, 9, 10]. A recent meta-analysis by Hung et al. found that patients taking steroids preoperatively were at an increased risk of adverse surgical outcomes, including infection, readmission, and reoperation, following orthopedic surgery [7]. One possible mechanism for this is the paradoxical increase in systemic oxidative stress with chronic steroid use, defined as greater than three weeks of use [8, 25]. Thus, oxidative stress prior to surgery may be contributing to the higher rates of postoperative complications in chronic steroid users, which may warrant the investigation of preoperative antioxidant use [26]. Additionally, glucocorticoids interfere with immune cell function and suppress the production of humoral factors involved in the inflammatory process [27]. These immunosuppressive effects may compromise the body’s ability to combat bacterial invasion and colonization at the surgical site, thereby increasing the risk for postoperative infections. This risk may be more pronounced in weight-bearing joints like the hip and knee, as there are generally fewer complications in TSA compared to total hip (THA) and total knee arthroplasty (TKA) and thus possibly a greater potential for infectious complications [28–32]. Additionally, the most prevalent microorganisms in periprosthetic joint infections (PJI) following THA and TKA are the high virulence *Staphylococcus aureus* and *Staphylococcus epidermidis* species, compared to the low virulence *Cutibacterium acnes* most frequently associated with TSA (33, 34). Additional research on chronic steroid use prior to TSA and other shoulder surgeries, as well as comparison of PJI rates across different joints, may help to better understand this relationship.

Importantly, only one of the studies included in the meta-analysis by Hung et al. assessed shoulder surgeries. Using a multivariable regression, Aziz et al. found chronic preoperative steroid use to be independently associated with major, minor, and infectious complications [35]. However, arthroplasty was excluded in this analysis, which limits the generalizability of these findings to TSA. Additionally, a recent meta-analysis investigating intra-articular corticosteroid injections prior to TSA reported an increased risk for PJI [37]. This suggests that while chronic oral steroid use may not be associated with infectious complications in TSA, particularly in the early postoperative period, preoperative corticosteroid injections could present a different risk profile due to localized immunosuppressive effects. Given these differences in infection risk associated with various forms of corticosteroid administration, further studies are warranted to explore the potentially distinct impact of oral versus parenteral steroid use on infectious outcomes in orthopedic surgeries, particularly TSA.

Several factors may contribute to the association between chronic steroid use and readmission following surgery. For example, chronic steroid users may have pre-existing conditions or characteristics other than those accounted for in the current study, such as reduced bone density and glucocorticoid-induced osteoporosis, which may predispose patients to postoperative fractures [7, 37]. Chronic steroid users also tend to have higher rates of opioid usage, which may have contributed to the higher rates of readmission given that opioid use-related hospital admissions increased 58-fold from 2008–2018 [38, 39]. However, the specific reasons for readmission were not analyzed in the present study. It is therefore unclear whether they were related to the surgery, chronic conditions, or other reasons, which is important to delineate in future studies on chronic steroid use and TSA outcomes. The shoulder surgeon should carefully consider these findings in the context of preoperative risk assessments in chronic steroid users, as the identification of chronic steroid use as an independent predictor of readmission suggests a vulnerability among these patients. Further, the absence of a significant association between chronic steroid use and infectious outcomes following TSA highlights the potential complexity of this relationship as it relates to TSA and possibly shoulder surgery in general. Altogether, this information adds to the sparse literature on chronic steroid use and TSA outcomes and emphasizes the importance of implementing and enhancing risk stratification protocols for chronic steroid-using patients undergoing TSA.

This study is not without limitations. First, the CPT code 23,472 does not differentiate aTSA from rTSA. As a result, we were unable to compare outcomes between the two approaches. Future studies should distinguish between the two approaches so that the shoulder surgeon can make well-informed treatment decisions. Second, the NSQIP database does not distinguish between indications for steroid use, which would be beneficial for surgeons in preoperative risk assessments of TSA patients using steroids preoperatively. Third, the NSQIP database only reports 30-day outcomes, so the present study could only speak to early-onset infectious outcomes rather than long-term outcomes and may under-represent the overall morbidity and mortality associated with chronic steroid use prior to TSA. This is particularly notable because *Cutibacterium acnes*, the most common pathogen in shoulder infection post-arthroplasty, may not present symptoms for two or more years postoperatively [34, 40]. Such infections are unlikely to be captured within the 30-day follow-up period utilized in this study, highlighting a limitation of the NSQIP database. Lastly, we did not distinguish between reasons for readmission and could thus only speak broadly on the impact of chronic steroid use on readmission following TSA.

## Conclusions

This retrospective propensity score matched analysis revealed no difference between chronic preoperative steroid users and non-steroid users in terms of reoperation rates, specific infectious outcomes, and infectious outcomes altogether. However, readmission was found to be independently associated with chronic preoperative steroid use. While the short follow-up time of 30 days was a limiting factor, these findings suggest that infection risk may not be heightened in aTSA/rTSA to the same extent as in other orthopedic surgeries. Rather, other complications necessitating readmission may be more likely for these patients in the early postoperative period. Additional studies with longer follow-up assessing infectious outcomes and readmission are needed so that shoulder surgeons can integrate this knowledge into pre- and postoperative care plans for patients with chronic steroid use prior to aTSA/rTSA.

## Data Availability

The datasets used and/or analyzed during the current study are available from the corresponding author on reasonable request.

## Abbreviations

TSA: Total shoulder arthroplasty
aTSA: Anatomic total shoulder arthroplasty
rTSA: Reverse total shoulder arthroplasty
THA: Total hip arthroplasty
TKA: Total knee arthroplasty
ACS-NSQIP: American College of Surgeons National Surgical Quality Improvement Program
CPT: Current procedural terminology
BMI: Body mass index
ASA: American Society of Anesthesiologists
COPD: Chronic obstructive pulmonary disease
OR: Odds ratio
CI: Confidence interval
SPSS: Statistical Package for Social Sciences
